# The Past, Present, and Future of a Human T-Cell Leukemia Virus Type 1 Vaccine

**DOI:** 10.3389/fmicb.2022.897346

**Published:** 2022-05-04

**Authors:** Joshua J. Tu, Victoria Maksimova, Lee Ratner, Amanda R. Panfil

**Affiliations:** ^1^Center for Retrovirus Research, Department of Veterinary Biosciences, College of Veterinary Medicine, The Ohio State University, Columbus, OH, United States; ^2^Comprehensive Cancer Center, The Ohio State University, Columbus, OH, United States; ^3^Division of Molecular Oncology, Department of Medicine, Washington University School of Medicine, St. Louis, MO, United States

**Keywords:** HTLV-1, vaccine, envelope, retrovirus, glycoprotein

## Abstract

Human T-cell leukemia virus type 1 (HTLV-1) is an oncogenic human retrovirus which causes a lifelong infection. An estimated 5–10 million persons are infected with HTLV-1 worldwide – a number which is likely higher due to lack of reliable epidemiological data. Most infected individuals remain asymptomatic; however, a portion of HTLV-1-positive individuals will develop an aggressive CD4+ T-cell malignancy called adult T-cell leukemia/lymphoma (ATL), or a progressive neurodegenerative disease known as HTLV-1-associated myelopathy/tropical spastic paraparesis (HAM/TSP). Few treatment options exist for HAM/TSP outside of palliative care and ATL carries an especially poor prognosis given the heterogeneity of the disease and lack of effective long-term treatments. In addition, the risk of HTLV-1 disease development increases substantially if the virus is acquired early in life. Currently, there is no realistic cure for HTLV-1 infection nor any reliable measure to prevent HTLV-1-mediated disease development. The severity of HTLV-1-associated diseases (ATL, HAM/TSP) and limited treatment options highlights the need for development of a preventative vaccine or new therapeutic interventions. This review will highlight past HTLV-1 vaccine development efforts, the current molecular tools and animal models which might be useful in vaccine development, and the future possibilities of an effective HTLV-1 vaccine.

## Human T-Cell Leukemia Virus Type 1 Background

### Epidemiology

Human T-cell leukemia virus type 1 (HTLV-1) was the first identified human retrovirus and is the only oncogenic human retrovirus known to date (Poiesz et al., 1980; Hinuma et al., 1981). An estimated 5–10 million individuals are infected with HTLV-1 worldwide (Gessain and Cassar, 2012). Pockets of endemic infection exist in Southwestern Japan, sub-Saharan Africa, parts of South America, foci in the Middle East, Australo-Melanesia, and the Caribbean. However, given the modes of viral transmission, lack of large population-based studies, and insufficient screening or prevention in many countries, the number of HTLV-1-infected individuals is likely much higher than the last published estimate from 2012. Indeed, the number of HTLV-1 infections is increasing in parts of the world, with new reports outside the historical endemic regions (Martin et al., 2018). A recent study found communities of Aboriginal people in Australia had 45% HTLV-1 sero-positivity (Einsiedel et al., 2016). HTLV-1 is the etiologic infectious agent of two highly aggressive diseases, adult T-cell leukemia/lymphoma (ATL) (Uchiyama et al., 1977; Poiesz et al., 1980; Yoshida et al., 1982) and HTLV-1-associated myelopathy/tropical spastic paraparesis (HAM/TSP) (Gessain et al., 1985; Osame et al., 1986; Matsuura et al., 2016; Enose-Akahata et al., 2017).

### Transmission

Retroviruses can infect cells as either cell-free virus particles or through cell-to-cell transmission. Several studies have demonstrated that HTLV-1 is a highly cell-associated virus that exclusively relies on cell-to-cell transmission to infect a target cell (Igakura et al., 2003; Majorovits et al., 2008; Mazurov et al., 2010). As such, HTLV-1 is most efficiently transmitted through (1) mother-to-child transmission (MTCT), (2) contaminated blood products, and (3) sexual transmission involving the transfer of bodily fluids. MTCT of HTLV-1 occurs primarily through breastfeeding, with few cases of infection *in utero* or during delivery. Several risk factors increase the chances of HTLV-1 MTCT including duration of breastfeeding, HTLV-1 proviral load in the mother’s blood and/or breastmilk, level of HTLV-1 antibody titer, HLA concordance, presence of disease in the mother, Strongyloides co-infection, and family income (Rosadas and Taylor, 2019). MTCT has been estimated to account for up to 30% of HAM/TSP cases and almost all ATL cases demonstrating that MTCT contributes disproportionately more to the global disease burden than other transmission routes (Bartholomew et al., 1998). In the absence of a preventative vaccine, one of the best approaches to eliminate risk and reduce HTLV-1-associated diseases is antenatal screening and avoidance of breastfeeding – two options which unfortunately are not widely available or feasible in many developing parts of the world.

HTLV-1 transmission can also occur through exposure to infected blood products such as in intravenous (IV) drug users, blood donations, and organ transplantation. Cases of transplant-derived HAM/TSP and ATL have been reported in many countries (Tajima et al., 2016; Moreno-Ajona et al., 2018; Nayar et al., 2018; Taylor, 2018; Motomura et al., 2019; Roc et al., 2019; Goto et al., 2020). In one such report, three recipients were monitored following organ transplantation from a HTLV-1-infected individual (not known to be positive at time of donation) (Cook et al., 2016). Despite early anti-retroviral treatments, HTLV-1 rapidly disseminated in each organ transplant recipient. Proviral load set points were reached within 6 weeks following transplantation and clonal expansion of infected cells was observed, indicating a high rate of infectious spread.

Sexual transmission of HTLV-1 is more efficient from men to women than women to men (Kajiyama et al., 1986; Roucoux et al., 2005). Infection is also enhanced by the presence of sexually transmitted diseases (syphilis, herpes simplex virus type 2), which are thought to cause ulcers, disruption of mucosal barriers, and recruitment of inflammatory cells (Paiva and Casseb, 2014). Other factors associated with increased HTLV-1 sexual transmission include the presence of Tax antibodies, a higher proviral load in peripheral blood lymphocytes, increased cervicovaginal or seminal secretions, age at first sexual encounter, unprotected sex, and the number of sexual partners.

### Genomic Structure

**FIGURE 1:**
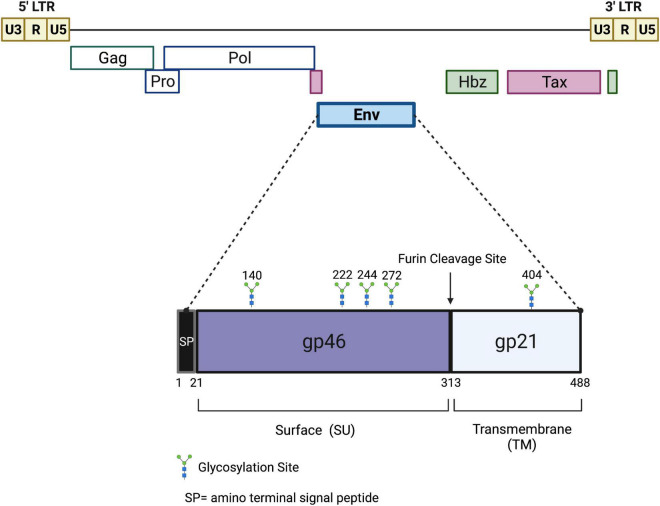
Schematic of the HTLV-1 proviral genome and envelope protein. Viral proteins Hbz, Tax, and Env are potential targets for a protective HTLV-1 vaccine. The viral *gag*, *pro*, *pol*, and *env* structural and enzymatic genes are flanked by 5′ and 3′ LTRs. The Hbz and Tax genes are located in the 3′ end of the viral genome. Hbz transcription initiates in the 3′ LTR. The envelope glycoprotein is comprised of two subunits, gp46 and gp21, which are cleaved at a furin cleavage site. Several potential glycosylation sites are denoted. Drawing is intended to be illustrative and not to exact scale.

Like all retroviruses, HTLV-1 has two positive polarity single-stranded RNA genomes, packaged with several viral enzymes (reverse transcriptase, integrase, and protease), surrounded by capsid proteins forming a roughly spherical viral nucleocapsid. A host-derived lipid bilayer envelope, studded with viral glycoproteins (gp62, Envelope, Env) surrounds the viral capsid. As a member of the deltaretrovirus genus, HTLV-1 is a complex retrovirus that expresses several regulatory and accessory genes, in addition to the standard structural and enzymatic genes common to all retroviruses (Figure 1). These regulatory and accessory genes are critical to numerous viral functions such as persistence, pathogenesis, transmission, and immune evasion (Bindhu et al., 2004; Bai and Nicot, 2012; Giam and Semmes, 2016). The viral genome is approximately 9 kb in length with long terminal repeats (LTRs) flanking the ends of the linear genome. The LTRs contain important elements for transcriptional regulation and polyadenylation of retroviral transcripts.

### Viral Entry and Lifecycle

In order to develop a protective HTLV-1 vaccine, the structure and function of the proteins that mediate viral entry and the viral life cycle must be characterized. The HTLV-1 envelope is the primary protein that mediates viral entry. It is an immunodominant viral glycoprotein composed of two subunits: gp46 (surface, SU) and gp21 (transmembrane, TM) (Figure 1). Like most retroviral envelope proteins, Env is synthesized as a precursor protein in the ER and then cleaved into gp46 and gp21 in the Golgi prior to transportation to the cell surface (Ghez et al., 2010). Experimental conditions which abolish precursor cleavage (tunicamycin or monensin treatments) results in the absence of envelope surface expression, indicating the importance of envelope cleavage during Golgi transport (Pique et al., 1992). Once at the cell surface, the gp46 portion of Env remains associated with gp21 through interchain disulfide bonds and the entire glycoprotein is anchored to the cell membrane through a membrane-spanning region found within gp21. On the surface of the virion, the gp46-gp21 subunits are organized as trimers and together facilitate viral entry: gp46 interacts directly with cell surface receptors and gp21 enables fusion of the viral and cellular membranes (Ghez et al., 2010). Prior to binding of gp46 to a receptor, gp21 is maintained in close association gp46 in a fusogenic-inactive metastable conformation (Jones et al., 2011). This conformation helps keep a fusion peptide found on gp21 buried, thus preventing premature membrane fusion, Env inactivation, and cell toxicity. gp21 activation occurs when gp46 binds to a cellular receptor, triggering alterations of the gp46/gp21 interaction and enabling gp21 to attain its fusogenic state (Weissenhorn et al., 2007; Melikyan, 2008). Based on a collective body of work, it is thought that Env uses neuropilin-1 (NRP1) and heparan sulfate proteoglycan (HSPG) for attachment and binding to target cells (Pinon et al., 2003; Ghez et al., 2006). The binding between gp46 and HSPG/NRP1 allows a conformation change in gp46 that exposes a binding domain that interacts with glucose transporter-1 (GLUT-1), followed by viral/cell fusion and the release of HTLV-1 nucleocapsid into the cytoplasm (Manel et al., 2003). The HTLV-1 Env glycoprotein also contains five potential N-glycosylation sites. Inhibitors that block Golgi glycosylation result in Env transport to the cell surface, but a reduction in syncytia formation indicating some defect in normal envelope function (Pique et al., 1992). HTLV-1 primarily infects CD4+ T-cells, but due to its widely available target receptors (NRP1, HSPG, GLUT-1), it has the ability to infect a wide variety of cell types such as CD8+ T-cells, B-cells, endothelial cells, myeloid cells, and fibroblasts (Furuta et al., 2017).

Unlike many other viruses, cell-free infection by HTLV-1 is extremely inefficient (although HTLV-1 infected cells do produce low titers of detectable virus) (Mazurov et al., 2010; Kalinichenko et al., 2022). Efficient HTLV-1 infection requires cell-to-cell contact between an infected cell and an uninfected target cell. One of the primary mechanisms for HTLV-1 transmission is thought to involve the formation of a virological synapse (VS), a specialized viral-induced cellular structure (Igakura et al., 2003; Majorovits et al., 2008). Once an HTLV-1 virion enters a target cell, its RNA genome is reverse transcribed into dsDNA. The dsDNA genome is then transported into the nucleus where it integrates into the host genome and is now referred to as a proviral genome. Once integrated, the proviral genome can be transcribed by cellular RNA polymerase II to express viral gene products.

Viral transcription initiates at promoter/enhancer elements found in the 5′ LTR, while a polyadenylation signal is found in the 3′ LTR. The first viral transcript expressed from the provirus after integration is the doubly spliced tax/rex transcript (Li et al., 2009). Tax functions as a viral transcriptional activator – it associates with the 5′ LTR at regions known as Tax-responsive elements (TREs) (Felber et al., 1985; Yoshimura et al., 1990; Zhao and Giam, 1992). Tax recruits the transcription factor CREB and facilitates its binding to the TREs (Zhao and Giam, 1991, 1992). Additional factors p300 and CBP are also recruited and form a complex at the 5′LTR which promotes transcription of all sense viral gene products (Giebler et al., 1997; Georges et al., 2003; Kim et al., 2007). Derived from the anti-sense strand of the proviral genome, Hbz can counteract several functions of Tax. Hbz interacts with CBP and p300, sequestering them away from Tax and thus diminishing Tax-mediated viral transcription (Clerc et al., 2008). Not only do Tax and Hbz play critical roles in viral transcriptional regulation, but they both play a critical role in viral pathogenesis.

In addition to viral transactivation, Tax is able to activate other cellular signaling pathways such as CREB, NF-κB, and AP-1 pathways (Giam and Semmes, 2016). Irregular activation of these pathways helps to drive clonal proliferation and survival of HTLV-1-infected T-cells. In addition to its role as a transcriptional activator, Tax serves as a viral oncoprotein and is critical for virus-mediated transformation. The Tax oncoprotein is also able to deregulate the cell cycle through silencing of cellular checkpoints that normally guard against DNA damage and chromosomal segregation. The cumulative effect of these aberrant cellular processes allows HTLV-1 to maintain a persistent viral infection through clonal expansion of infected T-cell clones, rather than primarily *de novo* infection. In addition to its transcriptional role, Hbz also regulates genomic integrity, apoptosis, autophagy, and immune evasion in infected cells (Matsuoka and Mesnard, 2020). Somewhat surprisingly, Hbz has been shown to promote proliferation of infected cells in both its protein and mRNA forms (Satou et al., 2006). The field has gradually adopted the idea that Tax is responsible for initiating transformation, while Hbz is responsible for the maintenance of infected cells.

### HTLV-1-Associated Diseases

It is estimated that HTLV-1 persists in approximately 10^3^–10^6^ infected T-cell clones within an infected host (Bangham et al., 2019). After initial viral infection, the virus is thought to become transcriptionally silent, or latent, as it is difficult to detect sense viral transcripts or proteins in infected individuals. However, the presence of activated cytotoxic T lymphocytes (CTLs) directed against sense viral antigens would suggest that some viral transcription is present, albeit at varying times and levels in HTLV-1-infected individuals. In fact, a recent study found that Tax is transcribed in intense, intermittent bursts within the infected cell (Billman et al., 2017). This burst is triggered by cellular stress and can be modulated through hypoxia and glycolysis (Kulkarni et al., 2017). Unlike Tax, Hbz is transcribed at more constant, lower levels. Indeed, hbz transcript is found in all asymptomatic, ATL, and HAM/TSP patient samples (Satou et al., 2006; Saito et al., 2009).

Unlike other oncogenic retroviruses, HTLV-1 does not capture a proto-oncogene or induce proviral insertional mutagenesis. Instead, the virus uses the combined effects of Tax, Hbz, and other virally encoded accessory proteins to induce cellular transformation. As such, disease development related to HTLV-1 infection can take upwards of several decades. HTLV-1 is the causative infectious agent of both an aggressive and fatal non-Hodgkin’s CD4+ T-cell lymphoma called ATL and a chronic, progressive neurodegenerative disease termed HAM/TSP (Uchiyama et al., 1977; Poiesz et al., 1980; Yoshida et al., 1982; Gessain et al., 1985; Osame et al., 1986). The lifetime risk of disease development related to HTLV-1 infection is 5–10%. HTLV-1-infected T-cells are the precursors to both ATL and HAM/TSP. Regulation of HTLV-1 gene expression allows the virus to evade immune detection, immortalize infected target cells, and establish persistent infection. There is limited information concerning the detailed molecular mechanism(s) behind disease development and disease penetrance. ATL is a heterogenous disease, posing a challenge to the selection of effective treatment strategies, and patients most commonly present with aggressive subtypes and face poor prognosis due to factors such as large tumor burden and lack of efficacious therapeutic drugs. Likewise, an ideal therapeutic strategy against HAM/TSP is not established, with most treatments aimed toward treating the symptoms and not the cause. Patients with HAM/TSP typically progress quickly after the onset of neurological symptoms, leading to deterioration in quality of life. The severity of both ATL and HAM/TSP—and limited treatment options for diagnosed individuals—highlights the need for development of a preventative vaccine or new therapeutic interventions.

### Immune Response to HTLV-1

Antibody and cell-mediated host immune responses against HTLV-1 occur within a few weeks after exposure to the virus (Kakuda et al., 2002; Bangham, 2003). During the first several months of infection, Gag antibodies are the most prevalent (Manns et al., 1991). Antibodies against Env appear shortly thereafter. In approximately half of all infected individuals there is a detectable Tax antibody response (Bangham, 2000). HTLV-1-infected patients often develop a strong CTL response with the majority of CTLs against Tax, and a smaller percentage directed against Gag, Env, and other non-structural gene products (Bangham et al., 1999). The immune response helps control viral infection but cannot eliminate the integrated virus. This immune pressure results in little to no cell-free HTLV virions found in the plasma (Pique and Jones, 2012). As a result, the most reliable method used to quantify viral infection or viral burden in both humans and animal models is proviral load. Proviral load is the number of proviral copies per set number of peripheral blood mononuclear cells (PBMCs). Higher proviral loads are associated with an increased risk of HTLV-1 disease. The immune response typically plays an important role in reducing proviral load in healthy HTLV-1-infected individuals.

Several studies have suggested the immune response can determine the course of HTLV-1-associated disease. The development of ATL is observed more frequently in patients who acquired HTLV-1 early in life (e.g., via breastfeeding) (Uchiyama et al., 1977; Fujino and Nagata, 2000). ATL is characterized by a late onset, and it is believed the initial reduced anti-viral immune response favors viral persistence. HAM/TSP patients typically acquire their HTLV-1 infection via infected blood products (Osame et al., 1990). This elicits a vigorous immune response, and these patients have 10–100-fold higher proviral load than asymptomatic patients (Bangham, 1993; Nagai et al., 1998). The humoral and cell-mediated CD4+ and CD8+ T-cell responses are highest in HAM/TSP compared to ATL and asymptomatic carriers (Usuku et al., 1988; Kamihira et al., 1989; Nagasato et al., 1991; Elovaara et al., 1993). Indeed, a vigorous expansion of CD8+ T-cells, the presence of Tax-specific CTLs in the cerebral spinal fluid (CSF), and high levels of anti-HTLV-1 antibodies in the serum and CSF characterize HAM/TSP (Enose-Akahata et al., 2017). There has been a suggested role of Tax-specific CTLs in the cellular destruction and inflammation in the central nervous system and spinal cord of HAM/TSP patients.

## HTLV-1 Animal Models Useful for Vaccine Research

The availability of a well-established animal model is an essential feature of vaccine design. The HTLV-1 field has two widely available and established animal models: one that mimics early infection in humans (rabbits) and one that models lymphoproliferative disease after HTLV-1 infection [humanized immune system (HIS) mice].

The rabbit model of infection was first demonstrated in 1985 when intravenous inoculation of rabbits with a HTLV-1-infected rabbit lymphoid cell line (Ra-1) lead to rabbit seropositivity (Miyoshi et al., 1985). Other possible routes of HTLV-1 transmission have also been demonstrated using rabbits, including semen and breast milk (Iwahara et al., 1990), making this a valuable model for studying and better understanding viral transmission. The rabbit model was further refined in the mid-1990s with the use of molecular clones for HTLV-1. Infection of rabbits with a HTLV-1 molecular clone behaved similarly to previously characterized virus isolated from infected humans (Zhao et al., 1995; Collins et al., 1996). This discovery allowed researchers to analyze the importance of viral genes or even viral regulatory elements during *in vivo* infection (Collins et al., 1998; Ye et al., 2003; Silverman et al., 2004; Arnold et al., 2006; Xie et al., 2006; Martinez et al., 2019). New Zealand white rabbits inoculated with lethally irradiated HTLV-1-infected cells become persistently infected. The early rabbit humoral antibody responses against viral antigens Gag and Env mimic that of asymptomatic early viral infection in humans. Proviral load is readily detected in these animals as early as 2 weeks post-infection. Detection of viral transcripts is possible but does present a challenge as viral transcripts peak early after infection and then decrease (Kannian et al., 2012). Importantly, these animals do not develop disease, but do recapitulate viral persistence and reliably mimic early viral infection events. The rabbit model of infection has several advantages which make it an ideal candidate system for vaccine development including low cost and little maintenance, the presence of a functional immune system, and genetic diversity of outbred rabbits. As with any animal model, rabbits also have limitations that should be carefully considered, such as the absence of disease related to HTLV-1 infection.

Murine models have significantly contributed to our understanding of HTLV-1 pathogenesis and have provided a valuable tool for the testing of therapeutics. A modified humanized mouse model was first introduced in 2006: NOG [NOD/SCID/gammac(null)] mice were inoculated with human PBMCs followed by infection with HTLV-1-infected cells (Miyazato et al., 2006). This resulted in a readily detectable proviral load in both the human CD4+ and CD8+ T cells. In a separate approach, engraftment of PBMCs from HTLV-1-carriers was performed in NOG mice (Takajo et al., 2007). These mice were able to harbor HTLV-1-infected human cells and permit clonal proliferation of these cells. In 2010, a more humanized mouse model was reported using NSG mice (NOD.Cg-PrkdcscidIl2rgtm1Wjl/SzJ) (Banerjee et al., 2010). NSG mice are severely immunodeficient (lacking B and T-cells, as well as functional NK cells), but are able to be humanized by engraftment of human CD34+ hematopoietic stem cells (HSC), PBMCs, patient derived xenografts, or adult stem cells and tissues. In this study, NSG mice were inoculated with *ex vivo* HTLV-1-infected CD34+ hematopoietic progenitor and stem cells (HP/HSCs) and subsequently developed CD4+ T-cell lymphomas with elevated T-cell proliferation. In a 2011 report, NSG mice were reconstituted with CD34+ stem cells and subsequently infected with HTLV-1 (Villaudy et al., 2011). This study found proviral integration in thymocytes and increased proviral load throughout the course of infection. Activated human CD4+ and CD8+ T-cells were found in the thymus and spleen of infected mice. These mice also developed hepatosplenomegaly, lymphadenopathy, and lymphoma/thymoma after infection, with Tax expression present in all tumors. Further studies have found that HTLV-1-infected HIS-NSG mice reproduce several characteristics of chronic infection in humans, such as activation of CD8+ T-cells and proliferation of effector/memory CD4+ and CD8+ T-cells (Espindola et al., 2021).

Although HIS mouse models are able to reproduce HTLV-1-associated T-cell lymphomas, they do not accurately depict human immune responses against HTLV-1. Humoral immunity and CTL-mediated cytotoxicity play a critical role in controlling the proliferation or selection of HTLV-1-infected T-cell clones *in vivo*. One approach to develop better adaptive immune responses was reported in 2014. In this study, HIS mice were created by transplanting human CD133+ hematopoietic stem cells into the bone marrow cavity of NOG mice using an intra-bone marrow injection method (Tezuka et al., 2014). After HTLV-1 infection, these mice developed a large number of pathological features characteristic of ATL including hyperproliferation of CD3+ T-cells, clonal proliferation of CD25+ CD4+ T-cells, splenomegaly, and inflammatory hypercytokinemia. These mice also exclusively developed leukemia, whereas previous models of HIS mice developed lymphoma or thymoma. Most importantly, these mice developed an adaptive immune response against HTLV-1, including HLA-restricted CTLs against Tax and IgG antibodies specific to HTLV-1.

HIS mice inoculated with HTLV-1 consistently reproduce the three key stages of HTLV-1-induced tumorigenesis: persistent infection, chronic proliferation of CD4+ T-cells, and development of lymphoproliferative disease. Future HIS mouse studies aimed at development of an adaptive immune response will be useful to evaluate the efficacy of vaccine candidates to prevent viral expansion *in vivo*. Each animal model has limitations that should be carefully considered when interpreting data, and ultimately human trials are a necessity for a successful human vaccine.

## Development of a HTLV-1 Vaccine: Past Vaccination Efforts

HTLV-1 vaccine research began shortly after the discovery of HTLV-1 nearly four decades ago. Although there is currently no candidate HTLV-1 vaccine in clinical trial, the development of a successful vaccine is considered feasible. HTLV-1 vaccine approaches can be organized into several broad categories: HTLV-1 protein-expressing recombinant viruses such as recombinant vaccinia virus (rVV), protein vaccines, DNA vector vaccines, and peptide vaccines.

### Recombinant Vaccinia Virus

One of the first published HTLV-1 vaccine studies in 1987 used a recombinant vaccinia virus (rVV) that expressed the envelope glycoprotein in place of the hemagglutinin gene (Shida et al., 1987). One inoculation of this vaccine candidate induced antibodies in rabbits and had a reported protective effect against HTLV-1 infection. Although proviral detection methods at the time of this study were rudimentary, this initial study does lend positive support for the eventual development of a successful HTLV-1 vaccine. Subsequent studies constructed various attenuated rVV expressing Env glycoprotein and inoculated them in rabbits (Shida et al., 1988). The different recombinant viruses synthesized similar amounts of envelope proteins *in vitro*, but had varying antibody responses, suggesting that the capacity of various vaccinia strains to induce antibody titers *in vivo* may be affected by their growth rates in rabbits.

A subsequent study in 1995 used the entire envelope DNA sequence (sequence taken from a West African healthy HTLV-1-infected patient) and expressed it in a highly attenuated poxvirus vaccine vector (Franchini et al., 1995). New Zealand white rabbits were inoculated with this live recombinant vector and exposed to an HTLV-1 cell-associated virus challenge. Although this study showed initial protection against the virus up to 5 months after the last immunization, all animals challenged 5 months after immunization were subsequently infected. Additional studies using rVV with Env at the site of hemagglutinin found that this vaccine candidate protected 1/3 vaccinated rabbits, with the initial protected rabbit becoming infected upon virus rechallenge 12 weeks after the first challenge (Hakoda et al., 1995). This trial suggests that this vaccine candidate was incapable of introducing neutralizing antibodies.

HTLV-1 Env rVV vaccine trials have also been tested in cynomolgus monkeys (Ibuki et al., 1997). This recombinant virus was constructed with plasmid pSFB5 which contains the A type inclusion body promoter of cowpox viruses and five units of synthetic vaccinia virus 7.5 kDa early promoter. The Env sequence was flanked with segments of the vaccinia virus HA gene. This vaccine was successful at inducing long-term protective immunity as immunized monkeys had no detectible virus after challenge and had sustained neutralizing antibody activity 136 weeks after challenge. Upon virus re-challenge at 136 weeks, the immunized monkeys had no antigen or provirus detected in cultured PBMCs.

A recent rVV study used either Hbz or Tax as a target for host immune responses (Sugata et al., 2015). Vaccination with either rVV expressing Tax or Hbz induced specific T-cell responses in mice and rhesus monkeys. Interestingly, multiple boosters were needed to elicit the Hbz response, likely due to low immunogenicity of Hbz protein compared to Tax. The authors were also able to demonstrate a protective effect with the anti-Hbz CTLs in an Hbz-transgenic mouse model. This study was the first to demonstrate that Hbz could be a potential immunotherapy target for HTLV-1 diseases.

### Protein Vaccines

Protein vaccine approaches have also showed promising results. One study from 1990 immunized pig-tailed macaques with soluble proteins isolated from MT2 cells, an HTLV-1 transformed cell line (Dezzutti et al., 1990). After challenge using a simian T-cell lymphotropic virus type 1 (STLV-1) producing cell line, the vaccinated macaques generated a strong serological response compared to placebo vaccinated animals. Sera from both groups of animals recognized Gag and Env proteins after virus challenge, however the vaccinated animals reacted more strongly to Env proteins. The antibodies produced by both groups of animals had antibody-dependent, complement-mediated cytotoxic activity directed against both HTLV-1 and STLV-1-infected cell lines. Most importantly, immunized macaques had no detectable reverse transcriptase activity after STLV-1 challenge.

### DNA Vector Vaccines

In 1997, the first report of direct DNA inoculation with a plasmid encoding HTLV-1 envelope in BALB/c mice was reported (Grange et al., 1997). Protein boosts with gp62 Baculovirus recombinant protein were also utilized. This study found that protein boosts were necessary to generate a high antibody response in mice with neutralizing antibody titers. Subsequent studies by this group found the choice of vector is critical for the design of DNA vector vaccines (Armand et al., 2000). Inoculation of mice with a human desmin muscle specific promoter driving Env expression (DesEnv) elicited a higher humoral response with better neutralization properties than injection with a CMV promoter driving Env expression. Another study used a Tax-coding DNA vaccine in a rat model [F344/N Jcl-rnu/ + (nu/ +)], exploring its use as a therapy for ATL (Ohashi et al., 2000). This vaccine used a mutant Tax which lacked transforming ability and was successful at inducing CTL responses in immunized rats, thus reducing HTLV-1 transformed tumor growth.

A study in 2001 evaluated the immunogenicity and protective effect of a vaccine involving priming with a highly attenuated vaccinia virus NYVAC HTLV-1 vaccine, followed by a booster with HTLV-1 Env DNA in squirrel monkeys (Kazanji et al., 2001). Squirrel monkeys were vaccinated with HTLV-1 Env and Gag- expressing NYVAC and naked env DNA. One group was primed with NYVAC and boosted with env DNA while the other group was primed with env DNA and boosted with NYVAC. The DNA prime/NYVAC boost was the most protective, with all three immunized monkeys having no detectable virus. In this study, anti-Env antibodies and cell-mediated responses against Env and Gag were detected in all protected animals. This study demonstrated that an ideal HTLV-1 vaccine candidate – which should induce long-lasting neutralizing antibodies against HTLV-1 and a strong cell-mediated immune response – might be difficult to achieve with a single vaccine preparation.

### Peptide Vaccines

Peptide vaccines have also had some success for HTLV-1 vaccine purposes. One of the first peptide studies in 1992 used a synthetic peptide derived from an immunodominant external envelope region mapping within amino acids 242–257 (Lairmore et al., 1992). When tested in rabbits, this peptide elicited a strong antibody response to gp46, however these antibodies failed to inhibit HTLV-1-mediated cell fusion and immunized rabbits were not protected from HTLV-1 challenge. This could be because while HTLV-1 specific antibodies were elicited, neutralization responses were either low or undetectable. A subsequent study tested several peptides from Env as vaccine candidates in the rabbit model (Tanaka et al., 1994). These epitopes were recognized by antibodies which could neutralize HTLV-1 syncytia and inhibit transformation. Env peptides 190–199 and 180–204 elicited neutralizing antibody responses in rabbits. When challenged with live HTLV-1-producing MT2 cells, the peptide immunized rabbits had no detectable provirus in PBMCs over an extended period of time. N-linked glycosylation is a major mechanism used by viruses to minimize neutralizing antibody response. A study examining the influence of N-linked glycosylation on HTLV-1 Env peptide structure and immunogenicity was conducted in 1995 (Conrad et al., 1995). A peptide from 233 to 253 of gp46 was engineered to contain an *N*-acetylglucosamine at residue Asn244. Similar conformation between both the glycosylated and non-glycosylated peptides was observed and both chimeric peptides were highly immunogenic in rabbits – producing antibody titers within 3 weeks post vaccination. Human sera from HTLV-1-positive individuals were able to recognize both the glycosylated and non-glycosylated constructs. Together this data suggests glycosylation of HTLV-1 gp46 does not affect the conformational preference or stability of the glycoprotein nor alter immunogenicity.

One disadvantage of peptide-based vaccines is their poor immunogenicity, thus often requiring tailored immunization procedures and adjuvants to evoke immune responses. A previous study used a gp46 peptide sequence from amino acids 175–218 linked C-terminal by a four-residue motif (GPSL) turn to the promiscuous T-cell epitope of the measles virus fusion protein (Frangione-Beebe et al., 2000). This chimeric peptide elicited high titer antibodies in both mice and rabbits and these antibodies were capable of inhibiting HTLV-1-mediated cell fusion. However, rabbits were not protected from cell-associated viral challenge, suggesting this vaccine failed to elicit cell-mediated immune responses necessary to protect against cell-associated HTLV-1 infection. Subsequent peptide-based vaccine studies have used a variety of target peptide sequences including a multivalent peptide from Tax connected by double arginine residues (aa11–19, 178–186, 233–241) (Sundaram et al., 2003), a peptide with the coiled-coil structure from gp21 (aa347–374) fused to a promiscuous T-cell epitope from tetanus toxoid (Sundaram et al., 2004), and most recently a unique multi-epitope chimera from immunodominant HTLV-1 epitopes in Tax, gp21, gp46, and p19 Gag (Kabiri et al., 2018).

Much of the HTLV-1 vaccine research that has predominated the literature are preventative vaccines. In 2015, a pilot study (three patients) investigated the safety and efficacy of a Tax peptide-pulsed dendritic cell vaccine used to treat ATL patients (Suehiro et al., 2015). HTLV-1-specific CTLs play a critical role in regulating the expansion or proliferation of HTLV-1-infected cells. The CTL responses in some HTLV-1-infected individuals and most ATL disease patients is severely impaired, allowing for infected cell proliferation and elevated proviral load. All three ATL patients in this study demonstrated Tax-specific CTL responses which peaked at 16–20 weeks post vaccination. Two patients achieved partial remission within the first 8 weeks (one of these later achieved complete remission), while the third patient (whose tumor cells lacked Tax expression) maintained stable disease and later developed slowly progressive ATL disease.

## Development of a HTLV-1 Vaccine: Future Vaccination Efforts

The development of mRNA vaccines has become a promising prophylactic strategy against viruses. Immunization with encapsulated mRNA offers numerous benefits over conventional vaccines, including improved safety through the delivery of a non-infectious agent and ability to regulate *in vivo* half-life, as well as enhanced efficacy through modulation of stability and translation (Pardi et al., 2018). Although vaccines composed of HTLV-1 env-encoding constructs have demonstrated efficacy *in vivo*, mRNA circumvents the potential for an anti-vector response. The potential use for mRNA vaccines in HTLV-1 research warrants study.

### HTLV-1 Vaccine Targets and Feasibility

An effective protective vaccine against HTLV-1 remains feasible for several reasons. One important argument can be found in the setting of MTCT. Babies born to HTLV-1-positive mothers have circulating anti-HTLV-1 antibodies at birth. These antibodies decrease exponentially during the first several months until most babies are seronegative by 6–9 months of age (Rosadas and Taylor, 2019). Not surprisingly, the duration of breastfeeding is one of the most important risk factors associated with MTCT of HTLV-1. Breastfeeding for shorter durations of time is associated with lower viral transmission rates, while breastfeeding for longer periods increases the risk of viral transmission (Rosadas and Taylor, 2019). This phenomenon has also been tested *in vivo* with rabbits. A HTLV-1 infected rabbit gave birth to 4 litters, 2 were given human HTLV-1 hyperimmunoglobulin (HTLVIG) and 2 were given no treatment at birth. HTLVIG treated litters had significantly less infection compared to control litters (8.3 vs. 42.9%) (Sawada et al., 1991). A separate study that infused rabbits with HTLVIG also showed protection against intravenous challenge (Takehara et al., 1989). However, a similar study in rats infused with anti-Env neutralizing antibody (LAT-27) observed a decrease in proviral loads when rats were challenged intraperitoneally but not orally, indicating that route of infection is important for *in vivo* studies pertaining to MTCT (Murakami et al., 2017). Taken together, these studies suggest that a vaccine that elicits a potent neutralizing antibody response may protect against infection, but also that the route of HTLV-1 infection should be considered in vaccine design.

The best way to prevent HTLV-1 infection is to block viral entry into host cells. The HTLV-1 envelope is one of the most immunogenic HTLV-1 proteins and is required for entry of target cells and establishment of initial viral infection. Antibody responses against Env can be detected in ATL patients as well as asymptomatic carriers (Enose-Akahata et al., 2012) and it is genetically stable and highly conserved among viral isolates both in its nucleotide and amino acid sequences (Sherman et al., 1993). Neutralizing antibodies have been mapped to epitopes in the portion of Env that mediates receptor binding, gp46, and have been shown to inhibit HTLV-1 Env-mediated entry of target cells (Kuroki et al., 1992; Astier-Gin et al., 1997; Kuo et al., 2011). The envelope protein alone has been shown to be immunogenic in animal models thus making it a good target protein for vaccine design.

Some of the major roadblocks to HIV-1 Env vaccine design are the extreme sequence diversity of viral isolates worldwide and the extensive glycosylation of HIV-1 Env. The HTLV-1 genome exhibits very few sequence variations, and a low overall genetic diversity is found in gp46 (Mota-Miranda et al., 2013). This is likely because HTLV-1 persists through clonal expansion, rather than continual viral spread like HIV-1. The glycosylation of HIV-1 Env has also been shown to change the exposure of neutralizing antibody epitopes, creating an additional hurdle for vaccine design. Conversely, HTLV-1 Env is not heavily glycosylated (5 potential sites) compared to HIV-1 Env (∼25 potential sites). In fact, *in vivo* data in rabbits suggests glycosylation of HTLV-1 gp46 does not alter immunogenicity (Conrad et al., 1995).

Other viral proteins which have been targeted in HTLV-1 vaccine studies are Tax and Hbz. Since HTLV-1 infection primarily occurs through cell-to-cell transmission, it may be important to create cellular responses against viral proteins that are widely expressed and presented by infected cells. In the context of acute infection, Tax is highly expressed by infected cells. Additionally, several peptides made from the Tax sequence are immunogenic *in vitro*. However, it has been shown that Tax expression can fall to undetectable levels in ATL patients, making anti-Tax responses as an ATL therapeutic an ineffective strategy. However, Tax could still be a viable target for HAM/TSP therapeutic vaccines as well as for protective vaccines if sterilizing immunity cannot be achieved. Hbz plays a key role in pathogenesis and persistence and, unlike Tax, it is detectible in all HTLV-1-infected cells and most diseased states. This makes Hbz an interesting target for therapeutic vaccine design in ATL as well as HAM/TSP patients, although the feasibility of this strategy is somewhat unclear. One study identified an Hbz-specific CTL clone, thus demonstrating that the Hbz protein is immunogenic. However, the Hbz-specific CTL clone was unable to lyse ATL cells (Suemori et al., 2009). Another study also found limited killing from Hbz-specific CTLs compared to Tax-specific CTLs (Rowan et al., 2014). Conversely, mice vaccinated with recombinant vaccinia virus expressing Hbz induced CTL responses and increased survival in lymphoma cell-inoculated mice (Sugata et al., 2015). Additionally, in a human cohort with 30 participants, IL-2 secreting, CD8+ T cells specific for Hbz were associated with low viral load and an asymptomatic phenotype (Hilburn et al., 2011). The current data are somewhat inconclusive and more studies are needed to evaluate the feasibility of Hbz as a successful vaccine target. Interestingly, no studies have been performed testing the efficacy of a vaccine targeting both Hbz and Tax *in vivo*. Such a vaccine may be able to elicit higher CD4+ and CD8+ T cell responses than those targeting Hbz or Tax alone, and retain efficacy even if Tax is downregulated in HTLV-1 infected cells. Targeting multiple HTLV-1 viral proteins may be a promising approach for both therapeutic and preventative vaccines.

### Challenges to HTLV-1 Vaccine Development

Despite the feasibility of developing a protective HTLV-1 vaccine, no candidate vaccine has ever proceeded to a clinical trial. This is because there are still several challenges in vaccine research that need to be overcome to achieve a protective immune response in humans.

A major challenge in eliciting protective vaccine responses is the cell-to-cell transmission of HTLV-1. Cell-to-cell transmission of HTLV-1 can occur through the establishment of cellular conduits, the formation of a virological synapse (VS), or extracellular viral assemblies (Gross and Thoma-Kress, 2016). It is understood that HTLV-1 Env is required for cell-to-cell transmission, but all known infection mechanisms of the virus allow for immune evasion (Derse and Heidecker, 2003). While it has been shown that HTLV-1-infected patient sera do contain neutralizing antibodies that can inhibit cell-to-cell infection (Mazurov et al., 2010), it is unclear, at least *in vivo*, what neutralizing antibody titers need to be induced by a vaccine in order to be protective. Antibody effector functions like antibody-dependent cellular phagocytosis and antibody dependent cellular cytotoxicity (ADCP and ADCP), as well as cellular responses, may be required to identify and eliminate infected cells for protection.

Another challenge of HTLV-1 vaccine design is the lack of research characterizing the HTLV-1 Env structure. While HTLV-1 virions have been imaged and the structure of the gp21 transmembrane domain has been solved, the structure of subunit gp46 has still not been resolved (Kobe et al., 1999; Majorovits et al., 2008; Cao et al., 2015). Since this subunit of Env directly interacts with the host receptors required for infection, it is important that we visualize these receptor interactions for successful vaccine design. Previous studies have shown that HTLV-1 Env conformation is important for the proper binding of antibodies isolated from HTLV-1 infected patients (Frangione-Beebe et al., 2000). Protein imaging techniques like Cryo-EM have been used in the HIV-1 field to characterize broadly neutralizing antibodies (bnAbs) and have used this information to develop vaccine candidates that elicit bnAb precursors and robust neutralization responses in mice and Rhesus macaques (Saunders et al., 2019). Imaging has also been used in studying SARS-CoV-2, which allowed for the development of spike protein stabilized in the prefusion conformation used in Moderna’s mRNA vaccine, mRNA-1273 (Jackson et al., 2020; Wrapp et al., 2020). An understanding of the prefusion spike protein structure of SARS-CoV-2 has been essential to identify and characterize antibodies used in monoclonal antibody therapies (Baum et al., 2020; Hansen et al., 2020; Pinto et al., 2020; Loo et al., 2022). If the HTLV-1 field can image and solve the structure of the gp46 subunit, the possibilities for HTLV-1 vaccine design will increase significantly and accelerate the timeline for the development of HTLV-1 vaccine candidates.

## Conclusion

HTLV-1 is a bloodborne pathogen that infects 5-10 million people and transmission occurs through breastfeeding, sexual transmission, and contaminated blood products. It is the causative infectious agent of several diseases including an aggressive CD4+ T-cell malignancy (ATL) and a progressive neurodegenerative disease (HAM/TSP). Approximately 5–10% of infected individuals develop disease after a prolonged clinical latency period of several decades. Despite decades of research, the complex mechanism of HTLV-1 persistence and disease development remains poorly defined and current treatment options are largely ineffective.

There is currently no vaccine for HTLV-1, although the development of one is considered feasible. Past vaccination efforts have focused on preventative vaccines targeting the envelope glycoprotein. Various approaches including recombinant vaccinia virus vectors, protein vaccines, DNA vector vaccines, and peptide vaccines have been explored with some success. Env is one of the most immunogenic HTLV-1 proteins, and antibody responses against Env can be detected both in ATL patients and asymptomatic carriers. The genetic stability of Env, high sequence conservation among viral isolates, and anti-Env activity in infected individuals makes this protein a favorable target for vaccine development. Recent approaches have also examined the safety and efficacy of therapeutic Tax peptide-pulsed dendritic cell vaccines to treat ATL patients.

Current advances in vaccine research have made mRNA vaccines a promising prophylactic strategy against viruses, and their potential use for HTLV-1 is intriguing. While hurdles remain, such as viral transmission strategies (cell-to-cell) and lack of structural research tools, there are several advantages, including the wide use of animal models and highly conserved nature of the HTLV-1 genome, which would benefit the development of a preventative vaccine.

## Author Contributions

JT and AP wrote the first draft of the manuscript. VM and LR wrote sections of the manuscript. All authors contributed to manuscript revision, read, and approved the submitted version.

## Conflict of Interest

The authors declare that the research was conducted in the absence of any commercial or financial relationships that could be construed as a potential conflict of interest.

## Publisher’s Note

All claims expressed in this article are solely those of the authors and do not necessarily represent those of their affiliated organizations, or those of the publisher, the editors and the reviewers. Any product that may be evaluated in this article, or claim that may be made by its manufacturer, is not guaranteed or endorsed by the publisher.
